# Combination of Six Individual Derivatives of the Pom-1 Antibiofilm Peptide Doubles Their Efficacy against Invasive and Multi-Resistant Clinical Isolates of the Pathogenic Yeast *Candida* *albicans*

**DOI:** 10.3390/pharmaceutics14071332

**Published:** 2022-06-24

**Authors:** Michelle Häring, Valerie Amann, Ann-Kathrin Kissmann, Tilmann Herberger, Christopher Synatschke, Nicole Kirsch-Pietz, Julio A. Perez-Erviti, Anselmo J. Otero-Gonzalez, Fidel Morales-Vicente, Jakob Andersson, Tanja Weil, Steffen Stenger, Armando Rodríguez, Ludger Ständker, Frank Rosenau

**Affiliations:** 1 Institute of Pharmaceutical Biotechnology, Ulm University, Albert-Einstein-Allee 11, 89081 Ulm, Germany; michelle.haering@uni-ulm.de (M.H.); valerie.amann@uni-ulm.de (V.A.); 2 Max Planck Institute for Polymer Research Mainz, Ackermannweg 10, 55128 Mainz, Germany; tilmann.herberger@mpip-mainz.mpg.de (T.H.); synatschke@mpip-mainz.mpg.de (C.S.); kirschn@mpip-mainz.mpg.de (N.K.-P.); weil@mpip-mainz.mpg.de (T.W.); 3 Center for Protein Studies, Faculty of Biology, University of Havana, 25 Street, Havana 10400, Cuba; julio.perez@fbio.uh.cu (J.A.P.-E.); aotero@fbio.uh.cu (A.J.O.-G.); 4 Synthetic Peptides Group, Center for Genetic Engineering and Biotechnology, Havana 10600, Cuba; femvicente@gmail.com; 5 AIT Austrian Institute of Technology GmbH, Giefinggasse 4, 1210 Vienna, Austria; jakob.andersson@ait.ac.at; 6 Institute for Medical Microbiology and Hygiene, University Hospital Ulm, 89081 Ulm, Germany; steffen.stenger@uniklinik-ulm.de; 7 Core Facility for Functional Peptidomics, Ulm Peptide Pharmaceuticals (U-PEP), Faculty of Medicine, Ulm University, 89081 Ulm, Germany; armando.rodriguez-alfonso@uni-ulm.de (A.R.); ludger.staendker@uni-ulm.de (L.S.); 8 Core Unit of Mass Spectrometry and Proteomics, Faculty of Medicine, Ulm University, 89081 Ulm, Germany

**Keywords:** antimicrobial peptides, invasive clinical isolates, combination therapy

## Abstract

In previous studies, derivatives of the peptide Pom-1, which was originally extracted from the freshwater mollusk *Pomacea poeyana,* showed an exceptional ability to specifically inhibit biofilm formation of the laboratory strain ATCC 90028 as a model strain of the pathogenic yeast *Candida albicans*. In follow-up, here, we demonstrate that the derivatives Pom-1A to Pom-1F are also active against biofilms of invasive clinical *C. albicans* isolates, including strains resistant against fluconazole and/or amphotericin B. However, efficacy varied strongly between the isolates, as indicated by large deviations in the experiments. This lack of robustness could be efficiently bypassed by using mixtures of all peptides. These mixed peptide preparations were active against biofilm formation of all the isolates with uniform efficacies, and the total peptide concentration could be halved compared to the original MIC of the individual peptides (2.5 µg/mL). Moreover, mixing the individual peptides restored the antifungal effect of fluconazole against fluconazole-resistant isolates even at 50% of the standard therapeutic concentration. Without having elucidated the reason for these synergistic effects of the peptides yet, both the gain of efficacy and the considerable increase in efficiency by combining the peptides indicate that Pom-1 and its derivatives in suitable formulations may play an important role as new antibiofilm antimycotics in the fight against invasive clinical infections with (multi-) resistant *C. albicans*.

## 1. Introduction

**Figure 1:**
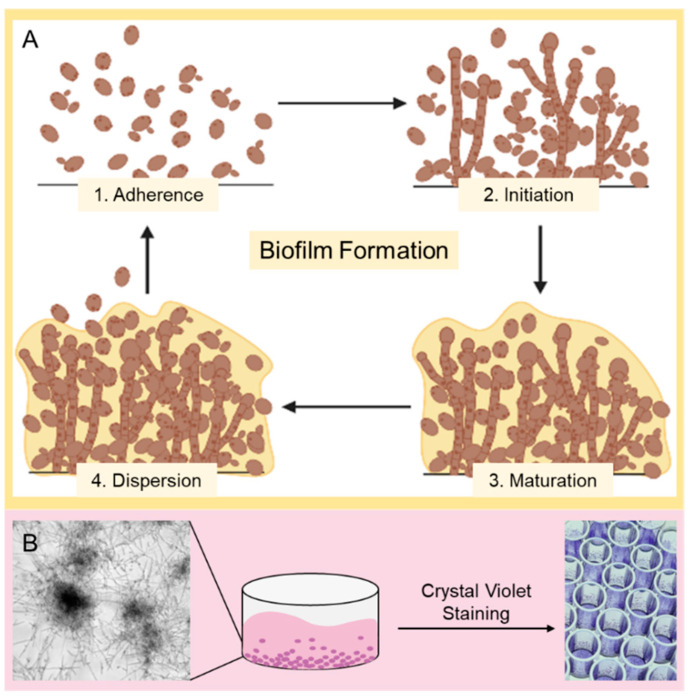
*Candida* biofilm formation, its typical appearance in microscopy and classic staining techniques. (**A**) Schematic overview of *Candida* biofilm formation. At first, (1. Adherence) planktonic yeast cells attach to the surface; after that (2. Initiation), the cells aggregate and proliferate until (3. Maturation) they form a mature biofilm with an extracellular matrix (ECM). In order to produce further biofilms, cells can detach themselves from the mature biofilm and find their way back into the planktonic phase (4. Dispersion). (**B**) Schematic representation of the experimental procedure. The efficiency of peptides on the biofilm (microscopy image with transmission light using a Leica Dmi8 (Leica Microsystems CMS GmbH, Wetzlar, Germany) in the lower left corner) of clinical *Candida* isolates was investigated using a microtiter dilution assay. During the incubation period, the *Candida* biofilm can form to different degrees depending on the potency of the peptides. After the incubation period, a crystal violet assay was performed to determine the biofilm mass. For this purpose, the biofilms were stained with crystal violet and then dissolved in 30% acetic acid to determine the biomass using a photometric measurement at 560 nm using a Tecan Infinite F200 microplate reader.

Invasive candidiasis is a fungal infectious disease referred to bloodstream and deep-seated infections caused by various *Candida* spp. with a mortality rate of up to 70% [1]. Yeasts of the species *Candida* are commonly present on human skin and in the gut microbiome and can be detected in 60% of healthy individuals [2]. These pathogens only become dangerous when there is an increased or abnormal colonization by these pathogenic cells [3]. Antifungal drugs such as echinocandins, azoles, and polyenes are widely used to treat such fungal infections [4,5]. Fluconazole is preferred as the first-line treatment option in certain clinical cases like endophthalmitis, meningitis, and urinary tract candidiasis [6]. Amphotericin B exerts strong and broad antifungal activity and is favored for external infections of the oral or vaginal mucosa as well as the gastrointestinal tract [7]. Intravenous administration in the case of systemic infections is possible but results in considerable side effects [8]. Although first-line drugs such as azoles and echinocandins are effective, the rapid development of intrinsic and acquired resistance to these agents is an increasing problem worldwide, caused by the high background usage of these compounds [6,9]. Drug resistance mechanisms differ depending on the type of the fungicide [10,11,12,13]. Resistance to amphotericin B is relatively rare and occurs mainly in the “superbug” *C. auris* [14]. Thus, in developing countries, these yeasts are among the top three pathogens causing bloodstream infections, with an incidence of 3–5 per 100,000 people [15]. In intensive care units, 1–2% of patients suffer from invasive candidiasis. In up to 95% of cases, these fungal infections are triggered by *Candida albicans* (*C. albicans*) [16,17]. Invasive candidiasis also favors long-term or repeated use of broad-spectrum antibiotics, which attenuates the intestinal microbiome and gives pathogenic yeasts a selective advantage over bacteria (specifically, clostridial Firmicutes and Bacteroidetes) that normally protect against this fungal infection [3,15]. In addition, breach of the marginal intestinal barrier due to chemotherapy, surgery, or central venous catheters, as well as iatrogenic immune suppression, can also cause translocation of *Candida* spp. from mucocutaneous sites into the bloodstream [1]. The ability of *C. albicans* to react to its environment with morphological changes (switching between unicellular cells to pseudohyphae and hyphae) represents a further challenge to the host defense mechanism, as the different morphotypes also have different surface compositions [18,19,20]. Spread of infection into the bloodstream is also enabled by the ability of yeast cells to adhere and invade endothelial and epithelial cells. In addition, the ability to adhere allows *Candida* spp. to form biofilms on biotic and abiotic surfaces [21,22]. Therefore, after adhesion of the yeast cells to a surface, proliferation and production of an extracellular matrix occurs, which leads to the formation of microcolonies. Detachment of these biofilm cells results in the formation of new biofilms, thus contributing to the further spread of the infectious disease (Figure 1) [23,24]. The cells acquire a significantly higher resistance to chemical and physical stress and increase the morbidity and mortality of infected patients after the formation of such biofilms and are a major cause of long-term candidemia and chronic infections of several pathogens [25,26,27]. Another important healthcare issue is biofilm formation on implant surfaces, which causes expenses of more than $3 billion every year in the United States alone [28]. Conventional therapies aim to treat *Candida* spp. infections by modifying the fungal cell membrane, but the yeast cells protect themselves by forming biofilms and can thus acquire higher-level resistance [22]. There is currently no dedicated biofilm-specific therapy that could stop the development of such resistance.

A promising class of drug molecules with a wide range of activity against viruses, bacteria, fungi, and parasites are natural and synthetic antimicrobial peptides (AMPs), also known as host defense peptides (HDPs) [29,30]. These oligopeptides consist of varying numbers of amino acids and are characterized by their secondary structure (α-helix, β-sheet, loops, extended) [31]. Conventional AMPs can act in two major ways. First, they can be membrane-active by integrating the peptide into the fungal membrane and forming different types of pores [32,33], and second, the effect can be intracellular by inhibiting important metabolic pathways in the pathogenic cell [34,35]. If the peptide is a “classic AMP”, i.e., it is membrane-active, pores are formed in the pathogenic membrane according to the barrel stave or the aggregate channel model. Another option is to aggregate on the membrane surface and prevent interactions of the pathogenic cell with its environment (carpet model) [36,37]. Various properties of the peptide are responsible for these modes of action. These include peptide length (to generate various structures, e.g., for amphipathicity) [38,39], net charge (to integrate with the negatively charged membrane) [40], helicity (to determine toxicity towards eukaryotic cells) [41], hydrophobicity (influence on activity and selectivity) [42,43], amphipathicity (also influences activity and selectivity) [44], and solubility (no aggregation and loss of function in an aqueous solution) [45]. Peptides with cationic properties can also interact with the negatively charged ECM of mature biofilms and thus exert potential antibiofilm activity [46]. They occur naturally in both eukaryotes and prokaryotes and are produced by specialized cells that are triggered by specific stimuli or produce them continuously. AMPs are part of the innate immune system and, as the first line of defense, play an important role in preventing infectious diseases [47,48,49,50]. The possible applications of these peptides are diverse; not only administration as an antimicrobial drug is conceivable [51], but also immobilization of such active substances on different surfaces in order to prevent the formation of a biofilm, for example, on implants [52]. There is also the possibility of combination therapies with conventional therapeutics [53] or conjugation of such AMPs to nanoparticles to combat resistant pathogens [54,55,56]. Another promising aspect is the supporting effect of AMPs in conventional antifungal therapy, as it was already shown that the combination of penicillin with pediocin and of ampicillin with nisin Z reduced the MIC against *Pseudomonas aeruginosa* by a factor of 13 and 155 [57]. Due to their mode of action, resistance to these agents is rare but not impossible. The transport of AMPs out of the cell by energy-dependent efflux pumps and modulation of intracellular targets like a resistance mechanism against other (unrelated) agents are also possible [58]. However, AMPs are an important and promising starting point for the development of new therapeutic approaches against multidrug-resistant pathogens, not only because of their excellent activity against many different pathogens, but also because of their almost unlimited natural occurrence and the possibility of synthesizing and optimizing them [29].

**Figure 2:**
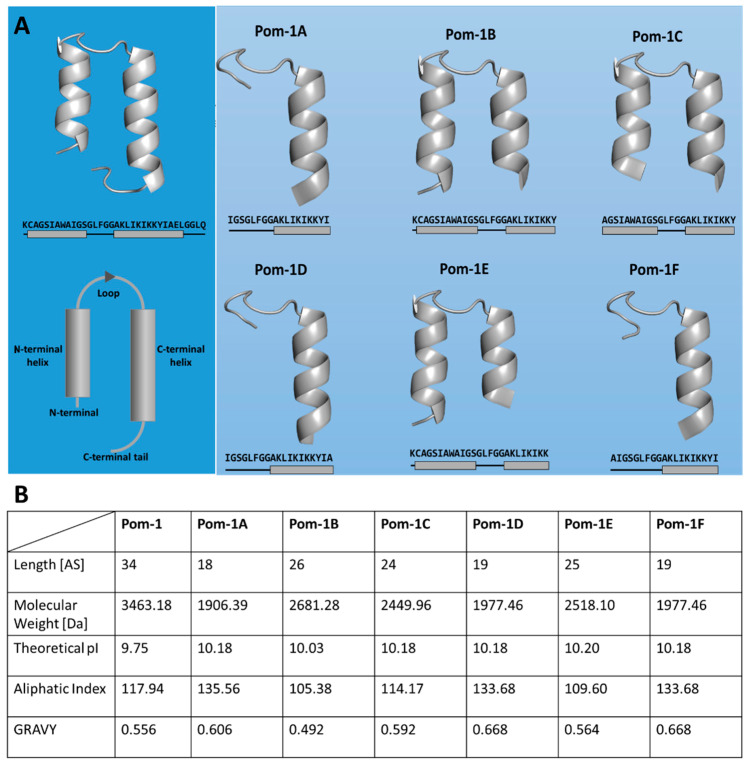
Antibiofilm peptides derived from the natural *P. poeyana* lead molecule Pom-1. (**A**) Illustration of the original AMP Pom-1 and its six derivatives (Pom-1A to Pom-1F) as ribbon models with QUARK and SwissModel, their amino acid sequences, and schematic representation of the α-helical structures. (**B**) Properties of Pom-1 and its derivatives calculated with ExPASy ProtParam [66]. Given are the theoretical isoelectric point (pI), the aliphatic index, and the grand average hydropathy index (GRAVY). These properties influence the mode and level of antimicrobial activity [31].

Mollusks represent interesting organisms for the identification of AMPs as they protect themselves exclusively with their innate immune system as they do not possess an adaptive immune system and thus have a wide range of AMPs [59,60]. The recently investigated AMPs Pom-1 and Pom-2 originate from this class of organisms and were first isolated from the Cuban freshwater snail *Pomacea poeyana* and then chemically resynthesized. These peptides showed antimicrobial activity not only against the bacteria *Pseudomonas aeruginosa*, *Klebsiella pneumoniae*, and *Listeria monocytogenes*, but also against planktonic cells and biofilm formation of various *Candida* species, and in addition, low cytotoxicity against human macrophages was observed [24,61]. Based on their antibiofilm mechanism of action, these and other snail-derived [24,62,63,64] peptides have been discussed to act not like conventional membrane-active peptides by forming pores, but by binding to the pathogenic membrane and thus preventing cell–cell and/or cell–substrate interactions, resulting in the prevention of biofilms [61]. This theory is supported by the fact that only a marginal reduction of viability of the *Candida* species and no significant toxicity towards human cells has been observed so far [61]. Due to the undoubtedly existing urge to develop new and potent antibiofilm medication strategies, further optimization of Pom-1 was approached with the aim of improving the specific antibiofilm activity by the generation of derivatives of this AMP (Figure 2) [65].

Based on the original study of the Pom peptides from *P. poeyana,* we previously showed that these six derivatives, designated Pom-1A to Pom-1F, significantly increased the antibiofilm activity against *C. albicans*, with Pom-1B, Pom-1C, and Pom-1D showing the highest improvement compared to Pom-1 as the lead structure (Pom-1D > Pom-1B > Pom-1C) [65]. However, this study was limited to the laboratory strain *C. albicans* ATCC 90028 as the model pathogen. The aim of this study was to demonstrate that this activity is also present against clinical isolates of *C. albicans* collected from patients suffering from invasive infections. Laboratory strains can be expected to differ from invasive isolates obtained from patients in clinical environments concerning biofilm formation and resistance against antifungal drugs. In this study, we showed as a follow-up that the peptides in fact also show remarkable activity against invasive clinical isolates, including strains with a strong resistance against fluconazole and/or amphotericin B. Application in mixtures increased both the efficacy and the efficiency of the peptides. The preparations were active against biofilm formation of all isolates with uniform efficacy, and the total peptide concentration could be halved compared to the original MIC of the individual peptides. Interestingly, low concentrations of the peptides were found to be active in combination with 50% of the standard therapeutic fluconazole concentration for fluconazole-resistant isolates as well, suggesting a synergistic effect of the peptides and fluconazole. Without having elucidated the reason for these synergistic effects of the peptides so far, both the gain of efficacy and the increase in efficiency by combining the peptides lead us to believe that Pom-1 and its derivatives in suitable formulations may play an important role as new antibiofilm antimycotics in the fight against invasive clinical infections with (multi-) resistant *C. albicans*.

## 2. Materials and Methods

### 2.1. Materials

Acetic acid, agar-agar, crystal violet, 3-(*N*-morpholino)propanesulfonic acid (MOPS), peptone, and yeast extract were obtained from Carl Roth GmbH (Karlsruhe, Germany). RPMI-1640 medium supplemented with L-glutamine was purchased from Thermo Fisher Scientific (Waltham, MA, USA). Fluconazole was obtained from Merck KgaA (Darmstadt, Germany), amphotericin B—from Carl Roth GmbH (Karlsruhe, Germany). Each of Dulbecco’s modified Eagle’s medium (DMEM), fetal bovine serum (FBS) (10% (*w*/*v*)), and penicillin–streptomycin (100 U*mL^−1^, 1% (*w*/*v*)), as well as Accutase^®^ and Eagle’s minimum essential medium non-essential amino acids (MEM NEAAs) were obtained from Life Technologies (Carlsbad, CA, USA). Phosphate-buffered saline (DPBS) was also sourced from Life Technologies (Carlsbad, CA, USA).

### 2.2. Methods

#### 2.2.1. Cultivation of *Candida* spp.

*C. albicans* (ATCC 90028) as laboratory strain was purchased from the IPK Laboratory of Medical Mycology. Clinical *C. albicans* isolates were provided from the patient samples sent to the Microbiology Department for diagnostic purposes. Strains were collected anonymously, and it is therefore not possible to assign the strains to patients. The accreditation number of the Microbiology Department is DIN EN ISO15189:2014 (DAkks). They were all cultured on Sabouraud dextrose agar (40 g/L glucose, 10 g/L peptone, 20 g/L agar, pH 5.6). For suspension cultures, individual colonies were inoculated in test tubes in 5 mL of RPMI-1640 supplemented with L-glutamine and grown at 37 °C with orbital shaking at 150 rpm for 16 h.

#### 2.2.2. Peptide Optimization

The derivatives Pom-1A to Pom-1F were designed by the Core Facility Functional Peptidomics of Ulm University led by PD Dr. Ludger Ständker as described before [65].

#### 2.2.3. Biofilm Formation and Crystal Violet Assay/Biomass Quantification

The antifungal effect of Pom-1A to Pom-1F on *Candida* spp. biofilm formation can be determined following the Clinical and Laboratory Standard Institute guidelines (M27-A3) [67]. For this, 2.5 × 10^3^ yeast cells were incubated in 200 µL of RPMI-1640 medium supplemented with L-glutamine, fluconazole, amphotericin B, and Pom-1A to Pom-1F. Incubation was performed on flat-bottomed polystyrene microplates with 96 wells (Sarstedt AG & Co. KG, Nümbrecht, Germany) for 24 h at 37 °C without shaking. The subsequent treatment with crystal violet was originally developed by George O’Toole for bacteria and adapted to *Candida* biofilms [68]. For this purpose, the planktonic phase was removed, and the wells were washed twice with 200 µL of demineralized water. The remaining biofilm cells were treated with 200 µL of 0.1% (*w*/*v*) crystal violet solution for 15 min. After removing the solution, they were washed again twice with 200 µL demineralized water, and the microtiter plates were dried for at least 24 h at 25 °C. The stain was dissolved with 200 µL of 30% acetic acid and transferred to a new plate after 15 min. The absorbance at 560 nm was measured using a Tecan Infinite F200 microplate reader (Tecan Group Ltd., Männedorf, Switzerland). The resulting data were evaluated against the untreated controls so that the efficacy of the agents could be determined.

#### 2.2.4. Cell Culture

For the experiments, adenocarcinomic human alveolar basal epithelial cells A549 [69] and human dermal fibroblasts (HDFs) were used [70]. Cultivation was performed in DMEM with FBS (10% (*w*/*v*), 15% (*w*/*v*)), MEM NEAAs (1% (*w*/*v*)), and penicillin–streptomycin (100 U*mL^−1^, 1% (*w*/*v*)) at 37 °C in an incubator containing 5% CO_2_.

#### 2.2.5. Passaging Adherent Cell Cultures

An appropriate medium (DMEM supplemented with 10% (*w*/*v*) FBS for A549, DMEM supplemented with 15% (*w*/*v*) FBS for HDFs) was preheated to 37 °C before passaging. The medium was removed from the culture flask, and 3 mL of Accutase^®^ were added. The cells with Accutase^®^ were incubated for 5–10 min until the cells acquired a round shape. To ensure complete cell detachment, the culture flask was slapped against the back of the hand. The desired number of cells was aliquoted into a new culture flask with the medium already provided. The cells were then incubated at 37 °C with 5% CO_2_.

#### 2.2.6. Viability Assays for Cell Cultures

A resazurin assay was used to detect the viability of the cells. Therefore, 2 × 10^4^ cells per well of a 96-well plate were incubated in 200 µL DMEM with additives at 37 °C and 5% CO_2_. The medium was removed, and 100 µL of the medium and 100 µL of a peptide solution (2.5 µg/mL, 25 µg/mL) were added. After incubation for 24 h at 37 °C and 5% CO_2_, 20 µL of a resazurin solution (0.15 mg/mL) were added into each well, and the cells were incubated again for 24 h at 37 °C and 5% CO_2_. Fluorescence measurement (excitation wavelength—535 nm, emission wavelength—595 nm) of the resulting resorufin was then performed using a Tecan Infinite F200 microplate reader (Tecan Group Ltd., Männedorf, Switzerland).

#### 2.2.7. ExPASy ProtParam

The peptide properties were determined using the ProtParam analysis tool (ExPASy) [66]. The calculation of the GRAVY value (grand average of hydropathicity) (Equation (1)) and the aliphatic index (Equation (2)) took place according to the following formulas:


(1) $$\mathrm{GRAVY}=\;\frac{\mathrm{sum of hydropathy values of all the amino acids}}{\mathrm{number of residues in the sequence}}$$

(2) Alipathic index = X(Ala) + a × X(Val) + b × [X(Ile) + X(Leu)]           

Amphiphilic index determination was carried out by the addition of the mole percentages (X) of the amino acids alanine (Ala), valine (Val), isoleucine (Ile), and leucine (Leu) considering the relative volume of valine side chains (a = 2.9) and Leu/Ile side chains (b = 3.9) of Ala.

## 3. Results

**Figure 3:**
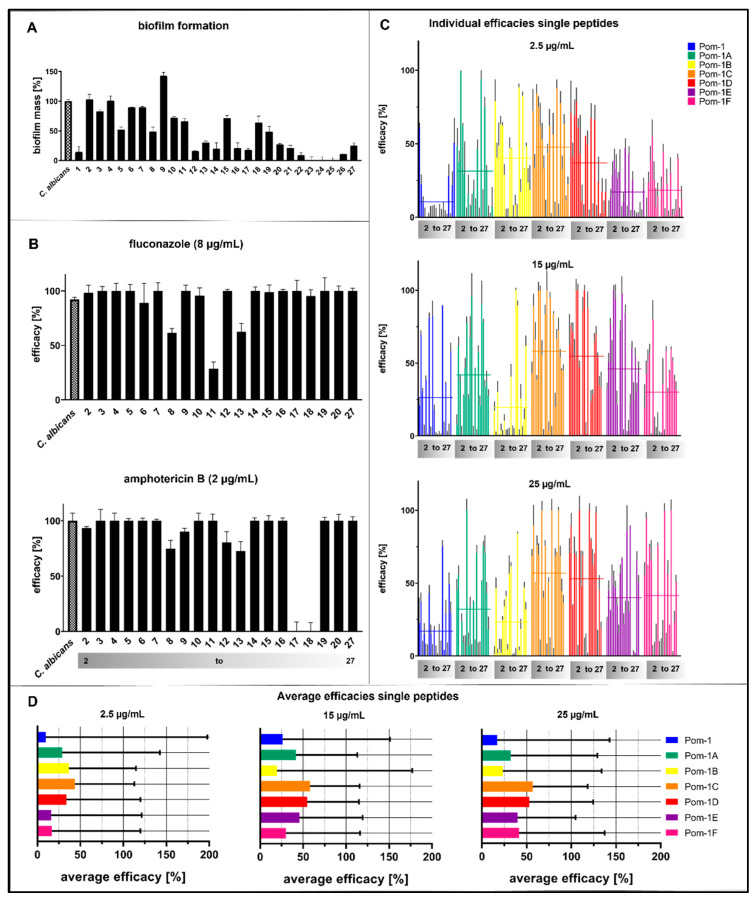
Detection of the biomass and antifungal activity of Pom-1 derivatives towards biofilm formation of invasive *C. albicans* clinical isolates by crystal violet assay. All the experiments were performed in triplicate, and the error bars depict the standard deviations. (**A**) Biofilm formation of *Candida* isolates without an agent. A laboratory strain of *C. albicans* was used as a reference. The *Candida* isolates were named with numbers. (**B**) Effect of fluconazole and amphotericin B on biofilm formation of the clinical isolates. The maximum inhibitory concentrations of 8 µg/mL for fluconazole and 2 µg/mL for amphotericin B were used. The *Candida* isolates were named with numbers. The grey bar was added to indicate the isolates to allow more transparency in the following figures (2–27). (**C**) Evaluated effects of 2.5 µg/mL (MIC), 15 µg/mL, and 25 µg/mL Pom-1A to Pom-1F on the biofilm mass of the clinical isolates. Each bar represents one isolate, repeated for each derivative. The mean values of the corresponding peptides are illustrated with horizontal lines. The evaluation of the individual peptides is continuously indicated with a grey color gradient. Pom-1 was tested as a control agent. A laboratory strain of *C. albicans* was used as a reference. (**D**) Summary of the average efficacies of Pom-1 and its derivatives based on (**C**). The average efficacies correspond to the mean values.

Invasive clinical isolates were collected from patients at Ulm University Hospital and initially subjected to an established biofilm formation assay based on cultivation in microtiter plates and subsequent analysis by the crystal violet assay originally published by O’Toole in 1998 for *Pseudomonas* biofilms [71], which we adapted for the analysis of *Candida* biofilms [24,62,65]. Of these 27 initial strains, 20 individual *C. albicans* isolates formed biofilms complying with the threshold that biofilm formation is regarded as significant when the biomass deposited on the plastic substratum of the microtiter plate exceeds 15% of the reference biofilms formed by the laboratory strain ATCC 90028 (Figure 3A). These 20 biofilm-forming isolates were then analyzed for resistance against therapeutically relevant concentrations of fluconazole and amphotericin B (8 µg/mL (*w*/*v*) and 2 µg/mL (*w*/*v*), respectively), and four isolates were found fluconazole-resistant, six—amphotericin B-resistant. Strains were considered resistant when the biofilm was detectable and inhibition of the antimycotics was lower than 100% (Figure 3B). Interestingly, isolates 8 and 13 were resistant to both compounds (Figure 3B). The peptide derivatives Pom-1A to Pom-1F were previously found to predominantly be active against *C. albicans* biofilms, with a minimal biofilm inhibitory concentration (MBIC) of 2.5 µg/mL (*w*/*v*), whereas planktonic growth was only inhibited moderately, and this inhibition was not improved by concentrations higher than 15 µg/mL (*w*/*v*) for the laboratory reference strain [65]. Considering clinical isolates potentially more robust and less sensitive against our peptide inhibitors compared to the reference strain, we decided to analyze biofilm inhibition with the minimal *C. albicans*-inhibiting concentrations of 2.5 µg/mL (*w*/*v*), 15 µg/mL (*w*/*v*), and 25 µg/mL (*w*/*v*) (i.e., 10× MBIC). The original Pom-1 peptide failed to inhibit 70% of the biofilms of these isolates at the MBIC (2.5 µg/mL (*w*/*v*)), whereas the number of strains affected by the peptides in biofilm formation was considerably increased with the derivatives (Figure 3C, 2.5 µg/mL (*w*/*v*)), which also had a higher efficacy, with Pom-1C in particular being the best derivative, with a fourfold average increase in efficacy (threefold for Pom-1B). However, even the best peptides resulted in an inhibition of less than 50%. The tenfold increase in peptide concentration (25 µg/mL (*w*/*v*)) only led to a nonproportional 10% increase in efficacy, indicating that this slight improvement was due to a drastic decrease in efficiency (Figure 3D). Nevertheless, Pom-1A completely inhibited biofilms of a single isolate (isolate 4) at the 2.5 µg/mL (*w*/*v*) MBIC. At the highest concentration of 25 µg/mL (*w*/*v*), all the peptides except Pom-1B and the original Pom-1 were 100% active against several individual strains (Figure 3C).

**Figure 4:**
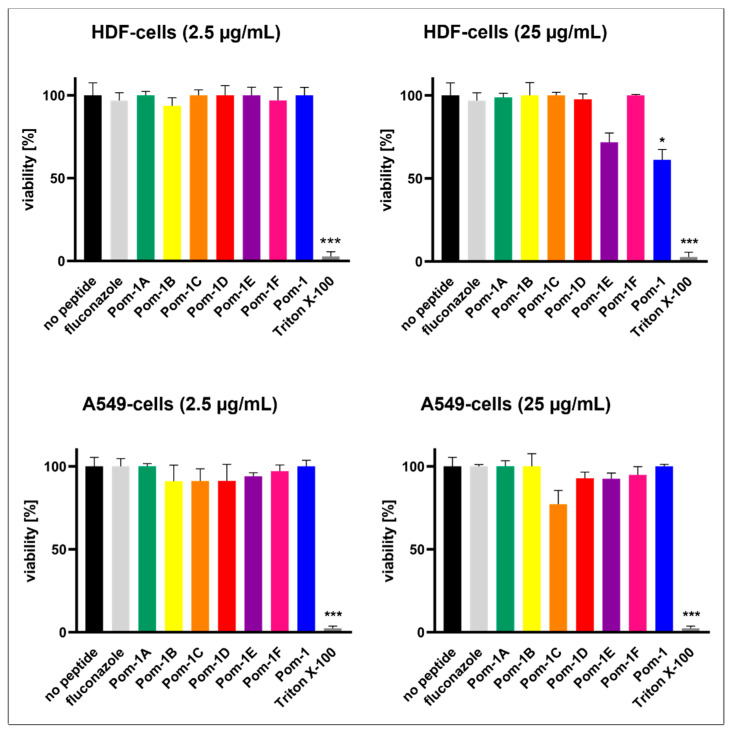
Cell viability of HDF and A549 cell lines without an agent and with Pom-1A to Pom-1F. Fluconazole and Triton X-100 were included as controls. All the experiments were performed in triplicate, and the error bars depict the standard deviations. Cell viability was tested after addition of 2.5 µg/mL and 25 µg/mL. Statistical analysis was performed with a *t*-test; *p*-values < 0.05 were considered significant (* *p* <0.05; *** *p* < 0.001). The columns without specific labeling show no significant differences (ns).

Apart from the lower efficiency of the peptides at 25 µg/mL (*w*/*v*), which already disqualified this concentration for the development of a therapy, it led to considerable cytotoxicity towards HDF cells for Pom-1E and the original Pom-1 peptide, whereas no significant effects on the viability of A549 cancer cells were observed. In contrast, at 2.5 µg/mL (*w*/*v*) (MBIC), none of the peptides, as well as fluconazole, were toxic neither to HDF nor A549 cells, whereas Triton X-100 as a known cell-lysing and thus toxic control perfectly worked to reduce cell viability to zero (Figure 4).

**Figure 5:**
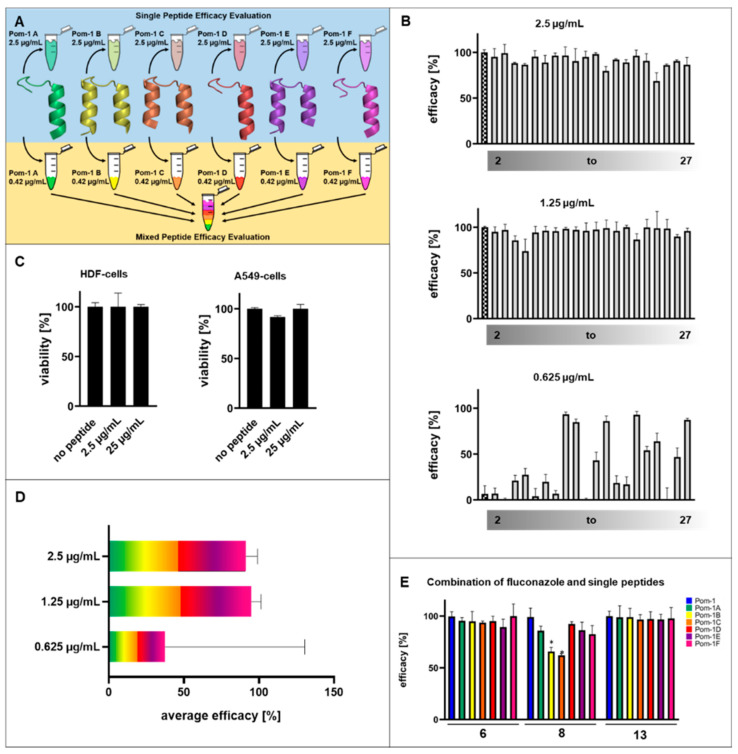
Antifungal activity of the combination of all Pom-1 derivatives on the biofilm formation of *Candida* spp. clinical isolates determined by crystal violet assay. The values of the laboratory strain of *C. albicans* were used as a reference. (**A**) Illustration of the step-by-step mixing procedure of the Pom-1 derivatives. As the first step, each Pom-1 derivative was analyzed separately on the *Candida* isolates (single peptide efficacy evaluation). This was followed by testing the combination of all the derivatives Pom-1A to Pom-1F using the MIC of 2.5 µg/mL in each case to obtain an equivalent mixture (mixed peptide efficacy evaluation). (**B**) Observed effect of 2.5 µg/mL, 1.25 µg/mL, and 0.625 µg/mL of the combination of Pom-1A to Pom-1F. Each bar represents one isolate, repeated for each derivative. The mean values of the corresponding peptides are illustrated with horizontal lines. The evaluation of the individual peptides is continuously indicated with a grey color gradient. The squared bars represent the control (*C. albicans* laboratory strain). (**C**) Cell viability of the HDF and A549 cell lines after addition of 2.5 µg/mL or 25 µg/mL of the combined Pom-1 derivatives. All the experiments were performed in triplicate, and the error bars depict the standard deviations. (**D**) Deduced average efficacy of the combination of the derivatives Pom-1A to Pom-1F based on (**B**), color code according to Figure 3C. The efficacy corresponds to the mean values. (**E**) Pom-1 and its derivatives enhance the fluconazole sensitivity of fluconazole-resistant *C. albicans* strains. The impact of each peptide variant was tested individually. A concentration of 4 µg/mL (*w*/*v*) fluconazole and 0.625 µg/mL (*w*/*v*) peptide was used. All the experiments were performed in triplicate, and the error bars depict the standard deviations.

Since the results of the individual peptides were not very promising therapeutically (Figure 5A “Single Peptide Efficacy Evaluation”, upper panel), we decided to test whether the individual peptides achieved improved efficacy in combination at the same concentration (Figure 5A “Mixed Peptide Efficacy Evaluation”, lower panel). The individual peptides Pom-1A to Pom-1F were mixed in equal amounts (0.42 µg/mL (*w*/*v*) per peptide) to give again the MBIC of 2.5 µg/mL (*w*/*v*) as an equally composed working solution. For this mixture, biofilm inhibition was considerably higher and resulted in an increase in affected isolates with a minimum inhibition of >70% with an average of >90% of all the strains tested, including the fluconazole- and amphotericin B-resistant candidates 6, 8, 9, 11, 13, 14, 17, and 18, demonstrating that this combination of peptides is also effective against multi-resistant *C. albicans* (Figure 5B,C). The cytotoxicity towards HDF and A549 cells, however, was not higher than that of the single peptides presented above, including the experiments with 10× MBIC (25 µg/mL (*w*/*v*)) (Figure 5D). To estimate the maximal efficacy potential, we decided to initially evaluate the minimal effective dosage by measuring two subsequent dilution steps resulting in the final mixed peptide concentrations of 1.25 µg/mL (*w*/*v*) and 0.625 µg/mL (*w*/*v*), representing 0.5× MBIC and 0.25× MBIC, respectively. The 0.5× MBIC experiments demonstrated that the efficacy was stable with all the isolates affected >70%, and the average efficacy was still >90% (Figure 5B, C). This constant efficacy is evidence of a doubling of the efficiency. In contrast, 0.25× MBIC resulted in a drastic decrease in the number of strains affected, and the average efficacy was accompanied by an enormous increase in data variability as indicated by the increase in the standard deviation of the average efficacy values. This indicates at least a considerable reduction in the robustness of the peptides in the mixture. However, the average efficacy of 0.25× MBIC still reaches the levels comparable to those of the single peptides used and to the concentrations of the original 1.0× MBIC (2.5 µg/mL (*w*/*v*)), again suggesting a substantial gain in efficacy as well as in efficiency when using the individual peptides in mixtures. Finally, we tested the opportunity to use the Pom-1 derivatives as agents that synergistically improve or restore the activity of fluconazole against the resistant isolates which lost the sensitivity against this classic antifungal compound. In this part of our study, the biofilm formation of the fluconazole-resistant isolates 6, 8, and 13 was tested in the presence of both fluconazole and the Pom-1A to Pom-1F peptides. Based on the 8 µg/mL (*w*/*v*) standard therapeutic concentration of fluconazole, which had turned out to be sub-inhibitory in our biofilm assay, and the information that 0.25× MBIC (0.625 µg/mL (*w*/*v*)) of the individual peptides) had failed, we tested whether this peptide concentration with a 50% concentration (4 µg/mL (*w*/*v*)) of fluconazole as the second antifungal compound would gain activity. Interestingly, we in fact achieved remarkable effects for all the peptides against biofilms of all the three isolates (Figure 5E). Biofilm inhibition was reduced to at least 60% for Pom-1C against isolate 8, whereas for isolates 6 and 13, the inhibition was close to 100% for all the combinations.

## 4. Discussion

One of the most common causes of hospital-acquired infections is candidiasis by different species, with alarmingly high mortality rates when these infections become systemic or even reach internal organs like liver, kidneys, or stomach [72]. *C. albicans* is the most prominent and prevalent pathogenic yeast in this context, since up to 90% of *Candida* infections originate from this microorganism [73]. It is widely accepted that up to 80% of *Candida* infections are associated with respective biofilms, which contribute to the high mortality rates and qualify this microbial life form as one of the main virulence traits associated with full pathogenesis of candidiasis [22]. In a sampling campaign at Ulm University Hospital, 27 biofilm-forming *C. albicans* isolates were obtained from invasive infections, four of which were found to be resistant to fluconazole, six—to amphotericin B, and two—to both classic antifungal compounds. The emergence of resistance has been recognized as a significant potential threat, particularly occurring in patients with AIDS and cancer after long-term treatment with immunosuppressive medications [74,75,76]. Since therapy of severe *Candida* infections has already been challenging due to the limited number of available antifungal drugs, the emergence of (multi-)resistant *C. albicans* poses a significant additional urgency for the development of alternative treatment options and potent novel antimycotics. AMPs have emerged as a promising class of alternative antifungal compounds and have been described to possess different modes of action with classic pore-forming peptides being the best established and best characterized subgroup. However, cytotoxicity is often a severe limitation of these peptides, and AMPs dedicated to inhibition of biofilm growth have been described [24,63,65]. In contrast to classic AMPs (e.g., LL37 [77]), Pom-1 and its derivatives have not only proven their potential to efficiently inhibit *Candida* biofilms while being only slightly active against planktonic cells [61], but combine these properties with the nontoxicity (except for Pom-1E, which was toxic to HDF cells at very high doses while exhibiting only marginal antibiofilm activity) towards human cells. This was discussed earlier as an indication for activity modes different from the typical simple pore formation and cell-killing activities [24,65], thus qualifying them as novel, truly biofilm-dedicated compounds. Although these results were already promising, it needed to be investigated whether the observed antibiofilm activity was also effective against biofilms of clinical invasive isolates of *C. albicans*. These were expected to differ in terms of their robustness, their capability to form biofilms, and, more importantly, could also be resistant to fluconazole or amphotericin B. Pom-1A to Pom-1F were active against most of the isolates, but the efficacy never exceeded 50%, accompanied by large standard deviations resulting from extremely different magnitudes of efficacy for individual isolates, with strains reacting perfectly to one of the peptides and not reacting to others. The peptide mixtures were not cytotoxic for HDF and A549 human cells at concentrations of 25 µg/mL as well. The mixture of Pom-1A to Pom-1F with the final concentrations equal to those in the single peptide analyses increased both the efficiency and the efficacy of the peptides since the concentration required to inhibit biofilm formation could be reduced to 50% while not only maintaining the efficiency, but also increasing the average inhibition as well as robustness of the activity, with more isolates being inhibited. Pom-1 and its derivatives were found not to belong to the group of AMPs with the classic mode of action and have been suspected to attack biofilm-relevant cell functions on the cell surface [65,78]. The observed synergistic enhancement of the individual peptides (at individually lower concentrations of each single peptide) strongly suggests that the Pom-1 derivatives may not act on distinct different effector epitopes like antiviral peptides, which affect the membrane fusion peptide of, e.g., the HIV [79], or most therapeutic neutralizing antibodies [80,81,82]. A strategy of pathogens is to use amyloid-like fibril structures to enhance the contact needed to express their full pathogenic potential [83,84,85,86,87]. *Candida* species have been found to use adhesins localized on the cell surface to form amyloidal fibril structures, which could be linked to the ability of pathogenic yeasts to form biofilms [88]. Without having systematically investigated the molecular targets of Pom-1 and its derivatives, we can speculate that microarchitectures on the cell surface and/or the structure of fibrillar materials present at early stages of biofilm development may represent a valuable reason for peptide activity and probable molecular targets. The synergism of AMPs represents a naturally evolved strategy to ensure host protection against a wide range of pathogens where the underlying mechanisms are elusive and not yet fully understood [89]. Often, enhanced antimicrobial activity is provided by AMPs of different AMP families synergistically protecting against a wide range of pathogens [90]. The underlying mechanisms of synergistic action of antibiofilm AMPs are still under investigation and have not been fully elucidated to date. The synergistic mode of action of antibiofilm AMPs may also be based on the fact that the different peptides complement or support each other in their mode of action and attachment [91,92]. Another possibility is the aggregation or interaction of the individual peptides into aggregates of higher structural complexity, which then may exert better antibiofilm/-microbial activity against the target pathogens than the individual peptides alone. This possible organization in supramolecular structures including the formation of amyloid-like fibrils or sheets needs to be investigated soon to elucidate the mechanism behind the improved activity of the peptide mixtures. As the combination of all six peptide derivatives had resulted in synergistic effects, we tested whether the peptides would also exert synergism with fluconazole. In fact, submaximal concentrations of the antifungal agent fluconazole (4 µg/mL (*w*/*v*)) also regained activity against the fluconazole-resistant *Candida* strains (at a concentration of 8 µg/mL (*w*/*v*) fluconazole) by mixing it with the 0.25× MBIC of the individual peptides Pom-1A to Pom-1F. The aim of a future combination therapy based on these results would, besides simply achieving synergism, also lie in avoiding the development of resistance. Such synergism has already been demonstrated with a combination of amphotericin B with the nucleobase derivative flucytosine in *Candida*, even in flucytosine-resistant strains [93,94]. We believe that in-depth understanding of cellular or extracellular targets and their interactions with Pom-1 and its derivatives will open valuable new perspectives to identify novel or further optimized (synthetic) peptide-based compounds as *Candida*-dedicated antibiofilm drugs.

## 5. Conclusions

Dedicated antibiofilm agents are an important issue, especially in the age of increasing microbial resistance development, not only to prevent biofilm formation, but also to possibly regain the activity of the standard antimicrobial therapeutics affected by the occurrence of resistance by posing additional selective pressure on the cells in the presence of the new compounds. The Pom-1 derivatives A–F, which had already shown strong antibiofilm activity against the laboratory strain ATCC 90028 of the pathogenic yeast *C. albicans* in earlier studies, were dramatically less effective against some resistant invasive clinical isolates of the same yeast species. However, combining all the six derivatives, the formation of their biofilms could be prevented efficiently. Moreover, the combination of fluconazole with the individual Pom-1 derivatives remarkably regained the sensitivity of invasive fluconazole-resistant isolates to a conventional fungicide.

## Data Availability

Not applicable.
